# Carbolic Acid in Cancer

**Published:** 1881-06

**Authors:** J. G. Westmoreland

**Affiliations:** Atlanta, Georgia


ATLANTA
Medical and Surgical Journal.

VOL. XIX.] JUNE—1881. [No. 3

### Original Communications.

### CARBOLIC ACID IN CANCER

BY J. G. WESTMORELAND, M. D., ATLANTA, GEORGIA.

Although carbolic acid has been known to the medical profession, and has been in general use for some time, yet its local effects and therapeutic results are very imperfectly understood. By some it is considered an escharotic, and applied with as much caution as nitric, chromic or other corrosive acids. Indeed few would think of applying the undiluted acid to diseased mucous membranes, for instance, without fear of destroying the entire tissue. Hence gynecologists who would gladly avail themselves of its local alterative or catheretic effect in intracervical and intra-uterine disease, look with horror upon the imaginary stenosis which is supposed to result from the destruction of the lining membrane and the consequent cicatricial tissue. This conclusion has, doubtless, been arrived at by the supposed analogy which exists between this and other mineral acids, without practical demonstration of the fact. Dr. V. H. Taliaferro, than whom we have no more experienced and better informed practitioner of this branch of medical science, does not hesitate to express such fears. It is therefore not surprising that those who have not attained to his eminence in the profession and his practical knowledge in the science and art of gynecology, should hesitate to make the application.

When the question of destruction by it of sound tissue, particularly that of mucuous membrane, is decided in the negative, (which it certainly will be by experience) and not until then, will carbolic acid assume its proper place in the materia medica. When it is known that the tendency to malignant, and non malignant ulceration, as well as ordinary chronic inflamation, may be arrested by its application, without the destruction of healthy structures beneath, this production of nature, separated from its crude connections by science and art, will begin fully to fill its destiny in the relief of human suffering and the cure of otherwise fatal disease. Progress in the use of this remedy is interfered with, not only by the error in regard to its destructive action upon sound tissues, but also by that of excessive pain following its application. This, doubtless, in part at any rate, originates in the fact that the delicate sound skin around the borders of ulcers and the skin connected with the outlet of mucous lined canals, such as the urethra, vagina, lips, etc. are made painful by contact with the acid, and also that a dilute preparation gives some pain, leaving the inference, according to the effect of other acids, that the undiluted remedy will give proportionately greater suffering. Although the mucous membranes are said to be a continuation of the cuticle, the latter is evidently more painfully and otherwise injuriously affected than the former. Upon tender portions of the skin, under the clothes vesication with burning pain, may result from a drop of undiluted acid, while no decided impression is made upon mucous tissue, except temporary pain.

It is also a singular fact that an inflamed mucous membrane, whether in a chronic or acute state is much less painfully affected than when in a healthy condition. In proof of this, reference may be had to the application in diphtheritic inflamation on the delicate mucous membrane of an infant's mouth, with but little if any more pain than is produced by a solution of chlorate potassium.

We have thought proper thus to preface our main subject with remarks touching the peculiarity of action and the safety with which carbolic acid may be used locally. In a previous article published in this journal the curative effect of the remedy in chronic inflammation of mucous membranes such as endomitritis etc. and diphtheria, was alluded to.

The various malignant or cancerous ulcerations, in the form of epithelioma, carcinoma, etc. prove to be not only a terror but scourge to the human race. Numerous suggestions have been made with reference to the extermination or removal of the germ or cell from the system. All have had their time and have passed out of notice, till now, the Chian turpentine has its day, and flattering indeed are the accounts of its wonderful cures. Shall this too have its brief period of apparent success and be unknown? Fain would we hope not. Would that statistics were multiplied to the eradication of all doubt of its permanent efficacy. No greater achievement can be had in the cause of humanity than the arrest and cure of cancerous disease.

Local applications in the form of escharotics, cauterants, catheretics, soothing demulcents and emollients have failed to accomplish the desired object. Arsenic and other catalytics have not sustained our hopes in the expulsion of the morbific cell.

Of Chian turpentine as a catalytic, general alterative or eradicator of the cancer cell by internal use, the writer knows nothing from experience, although much is claimed for it in this way. While we have not tested the internal use of carbolic acid for this purpose, judging from its peculiar local action in the relief of malignant tendency it is possible that benefit may be derived from its constitutional action, and to that end we propose a test of it the first opportunity that offers.

In a former article the partial history of two cases of supposed malignant uterine disease was given in which the acid was used. One was beyond all question cancerous ulceration, while the other exhibited the usual evidences of malignant cauliflower excresence of the os. These were treated almost exclusively with carbolic acid applied locally. The former improved rapidly in every respect until the case passed from under my care, and gave flattering hopes of permanent recovery, while the latter was perfectly relieved and has had no return of the disease.

Recently a case of epithelioma came under my care, in which a malignant ulcerous tumor existed on the face, involving the eye lids, cheek and side of the nose, to the extent of four or five inches. The whole tumor was removed by the knife and the eye ball extirpated.

Only a few days were required for the sprouting of malignant, fungous hemorrhagic granulations over the whole raw surface. The cut edge of the skin, which appeared free from disease at the time of operating, seemed particularly prolific of these unhealthy granulations, rising rapidly above the surface of the face. The application of pure crystalized carbolic acid, warmed to the liquid state, was commenced about the sixth day after the operation, and was continued daily, with the effect of leveling down the exuberant growth and destroying the red hemorrhagic appearance. In less than a week from the time of commencing the application the granulations were healthy, and the formation of new healthy skin proceeded from every direction around the large raw surface, except the upper portion of the nose, where a malignant tumefaction had all the time existed, extending across to a point near the inner canthus of the right eye. The application was made with a camel's hair brush, and to the fullest extent possible, without having the acid to ooze upon the sound skin; this being protected by a dry folded cloth kept near the raw surface, so as to absorb that which drained from the part to which it was applied. At the expiration of six weeks the raw surface was reduced to one third of the original size, and that which remained unhealed was in the direction of the unhealthy tumefaction on the nose. No healing or formation of new skin had occurred for a half inch in extent at this point. It was not found necessary, however, to apply the acid to all parts every day. At points where the surface kept smoothe and pale, it was not required more than two or three times a week, while the point referred to on the nose received daily applications in order to arrest exuberant growth and malignant tendency.

No pain at all is produced by contact of the acid with the diseased surface, but the lancinating pain which sometimes attends the disease is generally relieved by it. The amount of acid necessary may be determined by the visible effect had upon the part. The surface should be made completely white, and short of this we need not expect the full effect of the remedy.

In this case the diseased condition across the upper portion of the nose, and perhaps in the left nostril, may interfere with a perfect arrest of the disease, owing to the impossibility of applying directly to these parts. By vigilence, and following up the prolongations, however, it is not unreasonable to expect a permanent cure, notwithstanding the vast extent and long standing of the affection before the treatment was commenced.

